# The Branching Projection Pattern of Ventral Subicular Cells Revealing the Complex Integrative Role of Key Hippocampal Outputs in Driving Fear and Anxiety

**DOI:** 10.1002/hipo.70128

**Published:** 2026-09-20

**Authors:** Fernando Falkenburger Melleu, Karolina Domingues, Isadora D'Ávila Tassinari, Newton Sabino Canteras

**Affiliations:** ^1^ Department of Anatomy, Institute of Biomedical Sciences University of São Paulo São Paulo Brazil

**Keywords:** amygdala, anxiety, defensive behavior, fear, hypothalamus, neuroendocrine regulation, nucleus accumbens

## Abstract

The ventral regions of the hippocampus play a crucial role in emotional responses through projections to the hypothalamus. In this context, the ventral hippocampus sends extensive projections to components of the medial hypothalamic defensive circuit, where projections to the anterior hypothalamic nucleus (AHN) influence both anxiogenic and innate fear responses, as well as contextual fear reactions. This study aims to examine the branching projection pattern of hippocampal cells that directly project to the AHN and identify the complete range of brain regions affected by key hippocampal outputs that drive fear and anxiety. To accomplish this, we first explored the full range of hippocampal cells that project directly to the anterior hypothalamic area using an AAV retroviral vector carrying the enhanced yellow fluorescent protein (EYFP) reporter gene. We found that the hippocampal cells projecting to the anterior hypothalamus were located primarily along the entire rostro‐caudal axis of the ventral subiculum. Next, by combining an AAV retro vector injection into the AHN to insert Cre recombinase specifically in the subicular neurons projecting to the anterior hypothalamic region and subicular injections of an AAV vector expressing membrane‐bound GFP and the presynaptic protein synaptophysin fused to the red fluorescent protein mRuby in a Cre‐dependent manner, we investigated the collateral branching pattern of subicular neurons that project to the AHN. Our results demonstrated that ventral subicular neurons projecting to the anterior hypothalamus exhibit an extensive branching projection pattern that connects to several brain regions involved in various functions, including memory, motivation, arousal, circadian rhythmicity, neuroendocrine regulation, and goal‐oriented behavior. This reveals that ventral subicular anterior hypothalamic‐projecting cells display a widespread branching projection pattern, uniquely positioning them to influence complex, integrated responses while driving anxiety and fear behaviors.

**Abbreviations**
3v: third ventricle
ab: angular bundle
aca: anterior commissure
AcbC: nucleus accumbens, core division
AcbSH: nucleus accumbens, shell division
ADP: anterodorsal preoptic nucleus
AHN: anterior hypothalamic nucleus
AHNc: anterior hypothalamic nucleus, central part
AHNp: anterior hypothalamic nucleus, posterior part
alv: alveus
AMd: anteromedial thalamic nucleus, dorsal part
AMv: anteromedial thalamic nucleus, ventral part
AON: anterior olfactory nucleus
Aq: cerebral aqueduct
AVP: anteroventral preoptic nucleus
AVPV: anteroventral periventricular nucleus
BLA: basolateral amygdalar nucleus
BLAa: basolateral amygdalar nucleus, anterior division
BLAp: basolateral amygdalar nucleus, posterior division
BMAp: basomedial amigdalar nucleus, posterior part
BST: bed nucleus of the Stria terminalis
BSTal: bed nucleus of the Stria terminalis, anterolateral area
BSTam: bed nucleus of the Stria terminalis, anteromedial area
BSTav: bed nucleus of the Stria terminalis, anteroventral area
BSTdm: bed nucleus of the Stria terminalis, dorsomedial nucleus
BSTif: bed nucleus of the Stria terminalis, interfascicular nucleus
BSTmg: bed nucleus of the Stria terminalis, magnocellular nucleus
BSTov: bed nucleus of the Stria terminalis, oval nucleus
BSTpr: bed nucleus of the Stria terminalis, principal nucleus
BSTrh: bed nucleus of the Stria terminalis, rhomboid nucleus
BSTtr: bed nucleus of the Stria terminalis, transverse nucleus
BSTv: bed nucleus of the Stria terminalis, ventral nucleus
CA1: field CA1 of the hippocampus
CA3: field CA3 of the hippocampus
cc: corpus callosum
CeA: central amygdalar nucleus
Cg: cingulate cortex
CoAp: cortical amygdalar area, posterior part
cp: cerebral peduncle
CPu: caudateputamen
DG: dentate gyrus
DMH: dorsomedial hypothalamic nucleus
DP: dorsal peduncular cortex
DRN: dorsal raphe nucleus
ECT: ectorhinal cortex
ENTl: lateral entorhinal cortex
ENTm: medial entorhinal cortex
fi: fimbria
fx: fornix
ic: internal capsule
IL: infralimbic cortex
LA: lateral amygdalar nucleus
LDT: lateraldorsal tegmental nucleus
LHA: lateral hypothalamic area
LHAad: anterodorsal zone of the Lateral hypothalamic area
LHAai: intermediate zone of the lateral hypothalamic area
LHAjd: juxtadorsalmedial region of the lateral hypothalamic area
LHAjp: juxtaparaventricular region of the Lateral hypothalamic area
LO: lateral orbitofrontal cortex
LPO: lateral preoptic area
LS: lateral septal area
LSr: lateral septal nucleus, rostral part
LSv: lateral septal nucleus, ventral part
LV: lateral ventricle
mch: medial corticohypothalamic tract
ME: median eminence
MEAad: medial amygdalar nucleus, anterodorsal part
MEApd: medial amygdalar nucleus, posterodorsal part
MEApv: medial amygdalar nucleus, posteroventral part
MEPO: median preoptic Nucleus
MM: medial mammillary nucleus
MPA: medial preoptic area
MRN: mesencephalic reticular nucleus
MS: medial septal nucleus
NDB: nucleus of the diagonal band
och: optic chiasm
OT: olfactory tubercle
ot: optic tract
PA: posterior amygdalar nucleus
PAG: periaqueductal gray
PAGdl: periaqueductal gray, dorsolateral column
PAGdm: periaqueductal gray, dorsomedial column
PAGl: periaqueductal gray, lateral column
PAGvl: periaqueductal gray, ventrolateral column
PaSUB: parasubiculum
PBN: parabrachial nucleus
PH: posterior Hypothalamic nucleus
Pir: piriform cortex
PL: prelimbic cortex
PMd: dorsal premammillary nucleus
PMv: ventral premammillary nucleus
PoSUB: prosubiculum
PP: peripeduncular region
PPN: peduculopontine nucleus
PrSUB: presubiculum
PS: parastrial nucleus
PT: paratenial thalamic nucleus
PVH: paraventricular nucleus of the hypothalamus
PVp: periventricular hypothalamic nucleus, posterior part
PVT: paraventricular thalamic nucleus
RCh: retrochiasmatic nucleus
Re: nucleus reuniens
rf: fasciculus retroflexus
RSpv: retrosplenial area, anterior zone
SBPV: subparaventricular zone
SCh: suprachiasmatic Nucleus
Sfi: septofimbrial nucleus
SHi: septohippocampal nucleus
SNc: substantia nigra, pars compacta
SNr: substantia nigra, pars reticulata
sox: supraoptic decussation
SUBd: dorsal subiculum
SUBv: ventral subiculum
SUM: supramammilary nucleus
TEa: temporal associative cortex
TUM: tuberomammilary nucleus
VMH: ventromedial hypothalamic nucleus
VMHa: ventromedial hypothalamic nucleus, anterior part
VMHc: ventromedial hypothalamic nucleus, central part
VMHdm: ventromedial hypothalamic nucleus, dorsomedial part
VMHSh: ventromedial hypothalamic nucleus, shell
VMHvl: ventromedial hypothalamic nucleus, ventrolateral part
VO: ventral orbitofrontal cortex
VTA: ventral tegmental area
ZI: zona incerta

## 1 Introduction

Hippocampal circuits are organized along the dorsoventral axis (Fanselow and Dong 2010; Strange et al. 2014). Research has shown that lesions confined to the dorsal hippocampus impair memory, spatial navigation, and spatial learning (Strange et al. 2014). In contrast, lesions in the more ventral parts of the hippocampus affect emotional responses (Bannerman et al. 2002; Kjelstrup et al. 2002; Pentkowski et al. 2006). As a result, it is widely accepted that the dorsal regions of the hippocampus are primarily responsible for cognitive functions, particularly spatial memory, while the ventral regions are associated with emotional responses.

Of particular relevance, ventral hippocampal lesions have a significant impact on responses related to fear and anxiety. For instance, research conducted by Kjelstrup et al. (2002) found that selective lesions in the ventral hippocampus reduce defensive fear responses during exposure to the elevated plus maze. A different study showed that ventral hippocampal lesions affect not only spatial tasks but also non‐spatial tasks related to fear (Bannerman et al. 2002). This includes measures such as the time it takes to eat unfamiliar food and the level of social interaction in a familiar environment (Bannerman et al. 2002). Additionally, studies by Pentkowski et al. (2006) revealed that ventral hippocampal lesions significantly decreased innate fear responses during exposure to cat odor and impaired the ability to exhibit conditioned fear in response to cat exposure, cat odor, or footshocks.

The ventral regions of the hippocampus are believed to play a crucial role in emotional responses due to their denser connections with limbic‐related areas, such as the nucleus accumbens, amygdala, and hypothalamic regions that regulate autonomic, endocrine, and behavioral functions (Strange et al. 2014). Notably, the projections to the hypothalamus are particularly significant for mediating responses related to fear and anxiety. In this context, the ventral hippocampus sends extensive projections to components of the medial hypothalamic defensive circuit, which includes the anterior hypothalamic nucleus, the dorsomedial area of the ventromedial nucleus, and the dorsal premammillary nucleus (Gross and Canteras 2012). These components are involved in both innate and learned fear responses to predatory threats (Gross and Canteras 2012). Within the ventral hippocampus, the ventral subiculum is known to provide particularly extensive projections to the anterior hypothalamic nucleus and, to a lesser extent, to the cell‐poor region surrounding the dorsomedial part of the ventromedial nucleus while avoiding the dorsal premammillary nucleus (Gross and Canteras 2012). Research has shown that the ventral subiculum‐anterior hypothalamic nucleus (SUB–AHN) pathway, a glutamatergic projection (Yan et al. 2022), influences both anxiogenic and innate fear responses, as well as contextual fear reactions. The ventral SUB–AHN pathway drives avoidance behavior in anxious situations (Yan et al. 2022). Stimulation of this pathway triggers goal‐directed escape responses, and the optogenetic inhibition of the ventral SUB–AHN pathway impairs anxiety‐like avoidance behaviors and the retrieval of contextual memory when faced with a predator cue (Yan et al. 2022). Additionally, subicular influence on the medial hypothalamic circuit has been regarded as providing key information for the dorsal premammillary nucleus to function as a threat detector, sensing dynamic changes under threatening conditions as the animal approaches and avoids a potential threat (Viellard et al. 2024).

Recognizing the crucial role of the ventral SUB–AHN pathway in mediating defensive responses, this study aims to examine the projection patterns of hippocampal cells that directly connect to the AHN, an essential part of the medial hypothalamic defensive circuit. The objective is to identify the complete range of brain regions affected by key hippocampal outputs that drive fear and anxiety.

To accomplish this, we first investigated the full range of hippocampal cells that directly project to the anterior hypothalamic region. We found that these hippocampal‐anterior hypothalamic projecting cells were located primarily along the entire rostro‐caudal axis of the ventral subiculum. Next, we examined the projection pattern of anterior hypothalamic projecting cells throughout the entire rostro‐caudal extent of the ventral subiculum. The findings indicated that these cells display a widespread branching pattern, uniquely positioning them to influence complex, integrated responses while driving anxiety and fear behaviors. Therefore, it is likely that anterior hypothalamic ventral subicular‐projecting cells have a more global role, exerting broad influence over several brain regions that mediate a wide range of functions, including memory, motivation, arousal, and circadian rhythm, as well as neuroendocrine and goal‐oriented behavior control.

## 2 Methods

### 2.1 Animals

Thirty‐five male and seven female adult wildtype C57BL/6 mice and three female Ai6 mice (RCL‐ZsGreen, Jackson Laboratories), aged 3–4 months, B‐W: 25–30 g, were obtained from the breeding colony of the University of São Paulo Medical School (FMUSP) and used in the experimental procedures. Animals were housed in the Laboratory of Functional Neuroanatomy animal facility in groups of four per standard polycarbonate cage (30 × 20 × 13 cm; L × W × H). Cages were enriched with polycarbonate shelters (“mouse houses”), cardboard tubes to promote exploratory behavior, and nesting material composed of pressed cotton fibers. The cage floor was covered with standard wood‐chip bedding. Cages were cleaned, enrichment materials replaced (excluding shelters), and bedding changed three times per week. Mice were maintained in a temperature‐controlled environment (23°C ± 2°C) under a 12‐h light/dark cycle (lights on at 7:00 a.m. and off at 7:00 p.m.), with food and water available ad libitum. The animal facility was routinely monitored to ensure stable environmental conditions and animal welfare in accordance with institutional and international guidelines. All procedures were approved by the Committee on the Care and Use of Laboratory Animals of the Institute of Biomedical Sciences, University of São Paulo, Brazil (Protocol #8930240322). All efforts were made to minimize the number of animals used and to ensure their well‐being.

### 2.2 General Stereotaxic Surgery

Mice were anesthetized in an induction chamber saturated with isoflurane (Isoforine, Cristália Laboratories, SP, Brazil) and subsequently positioned in a stereotaxic apparatus (Kopf Instruments, CA, USA). Anesthesia was maintained with a continuous flow of 1%–2% isoflurane mixed with oxygen, and body temperature was kept constant using a thermostatically controlled heating pad. The scalp was shaved and disinfected, and a midline incision was made to expose the skull. Craniotomies were performed at the designated stereotaxic coordinates using a dental drill. Viral vectors were delivered using a 5‐μL Hamilton syringe (Neuros Model 7000.5 KH) connected to a motorized infusion pump (Harvard Apparatus). Injections were administered at a rate of 5 nL/min, and the needle was left in place for an additional 5 min following each infusion to minimize backflow of viral solution. After the surgical procedure, the incision was sutured, and mice were placed individually in a heat‐controlled cage for anesthesia recovery. After the recovery period, mice were returned to their cage after recovery from anesthesia.

### 2.3 Retrograde Tracing Experiments

For retrograde tracing experiments, animals were subjected to stereotaxic procedures as described above (*n* = 5 wildtype C57BL/6 male mice). Each animal received one injection of 50 nL of a viral vector solution with AAV retrograde serotype virus carrying the enhanced yellow fluorescent protein (EYFP) reporter gene (AAVretro‐CaMKII‐EYFP; Addgene: #50469; RRID: Addgene_50469; titer: ≥ 4 × 10^12^ vg/mL). Injections were made in the anterior hypothalamic region using the following stereotaxic coordinates: AP: −1.3; ML: 0.50; DV: −5.15. After a 4‐week period to allow EYFP expression, animals were euthanized as described below.

### 2.4 Anterograde Tracing of Subiculum>Hypothalamic Neuronal Branching Pattern

To evaluate the collateral branching pattern of subicular neurons projecting to the anterior hypothalamus (AH), animals were subjected to stereotaxic surgery as described above. We first injected a Cre recombinase expression vector with retrograde transport (AAVrg‐hSyn‐Cre‐WPRE‐hGH; Addgene #105553‐AAVrg; RRID: Addgene_105553; titer: ≥ 7 × 10^12^ vg/mL) into the AH, using the same volumes and coordinates for the hypothalamus as previously mentioned. Because this Cre vector does not contain a reporter gene, we added 2% fluorogold (1 μL) to the working aliquot to faintly label the hypothalamic injection sites. This resulted in a final viral vector titer of ≥ 5.6 × 10^12^ vg/mL. On the same surgical procedure, we then injected 80 nL of a Syn‐driven, Cre‐dependent expression of membrane‐bound GFP and the presynaptic protein synaptophysin fused to the red fluorescent protein mRuby (AAV5‐hSyn‐FLEx‐mGFP‐2A‐Synaptophysin‐mRuby; AddGene: #71760; RRID: Addgene_71760, titer: 7 ≥ 7 × 10^12^). Injections were made in three different subicular regions according to the results of retrograde tracing experiments, namely: (1) rostral ventral subiculum (*n* = 10 males and seven females wildtype C57BL/6 mice and *n* = 3 Ai6 female mice) (AP: −3.59; ML: −2.85; DV: −4.6); (2) Caudal ventral subiculum (*n* = 10 males wildtype C57BL/6 mice) (AP: −5.0; ML: −0.40; DV: −4.6); and (3) dorsal subiculum (*n* = 5 males wildtype C57BL/6 mice) (AP: −3.69; ML: −3.45; DV: −1.90). Moreover, a control group (*n* = 5) received only the AAV5‐hSyn‐FLEx‐mGFP‐2A‐Synaptophysin‐mRuby in the rostral ventral subiculum without retrograde Cre insertion from AAVrg‐hSyn‐Cre‐WPRE‐hGH injection into the AH.

This strategy yielded mGFP and the red protein mRuby expression only in subicular cells projecting to the anterior hypothalamic region, revealing the entire extent of the projection branching pattern of these cells. After 4‐week period, animals were euthanized as described below.

### 2.5 Perfusion, Tissue Processing, and Histology

Four weeks after the stereotaxic surgeries, animals were deeply anesthetized in an induction chamber saturated with isoflurane and transcardially perfused with 4% paraformaldehyde in 0.02 M phosphate‐buffered saline (KPBS; pH 7.4). Brains were removed and post‐fixed in the same fixative for 4 h at room temperature, then transferred to a 20% sucrose solution in 0.02 M KPBS at 4°C for 24 h for cryoprotection. Subsequently, brains were frozen and sectioned coronally at 30 μm using a sliding microtome. Sections were collected in five series, three of which were stored in cryoprotectant solution at −20°C until further processing.

### 2.6 Immunofluorescence

To amplify the GFP signal within viral‐labeled projection fibers, brain sections underwent an immunofluorescence protocol. Briefly, sections were recovered from storage and rinsed six times in 0.02 M KPBS for 5 min each. They were then immersed in a primary antibody solution and maintained at 4°C for 72 h. The primary antibody consisted of a chicken IgY anti‐GFP (Cat #A10262; RRID:AB_2534023; ThermoFisher, Waltham, MA, USA) diluted at 1:5000. After primary incubation, the tissue was incubated for 90 min with a fluorophore‐conjugated secondary antibody (AlexaFluor 488 goat anti‐chicken; Cat #A78948; RRID: AB_2921070; ThermoFisher, Waltham, MA, USA). The reaction was terminated by rinsing the sections in fresh KPBS. Following additional washes, sections were mounted on gelatin‐coated slides and coverslipped with Fluoromount (Sigma‐Aldrich). Fluorescent signals were visualized using a Nikon Eclipse 80i fluorescence microscope (Nikon Corporation, Tokyo, Japan) at ×4 magnification. Digital 8‐bit images (6000 × 3984 pixels, 300 dpi) were acquired in two channels (525 nm/630 nm) with a Nikon Digital Sight 10 CMOS camera controlled by Nis‐Elements v.6.10.01 (Nikon Corporation, Tokyo, Japan).

## 3 Results

### 3.1 Distribution of Hippocampal Cells Projecting to the Anterior Hypothalamic Region

**FIGURE 1:**
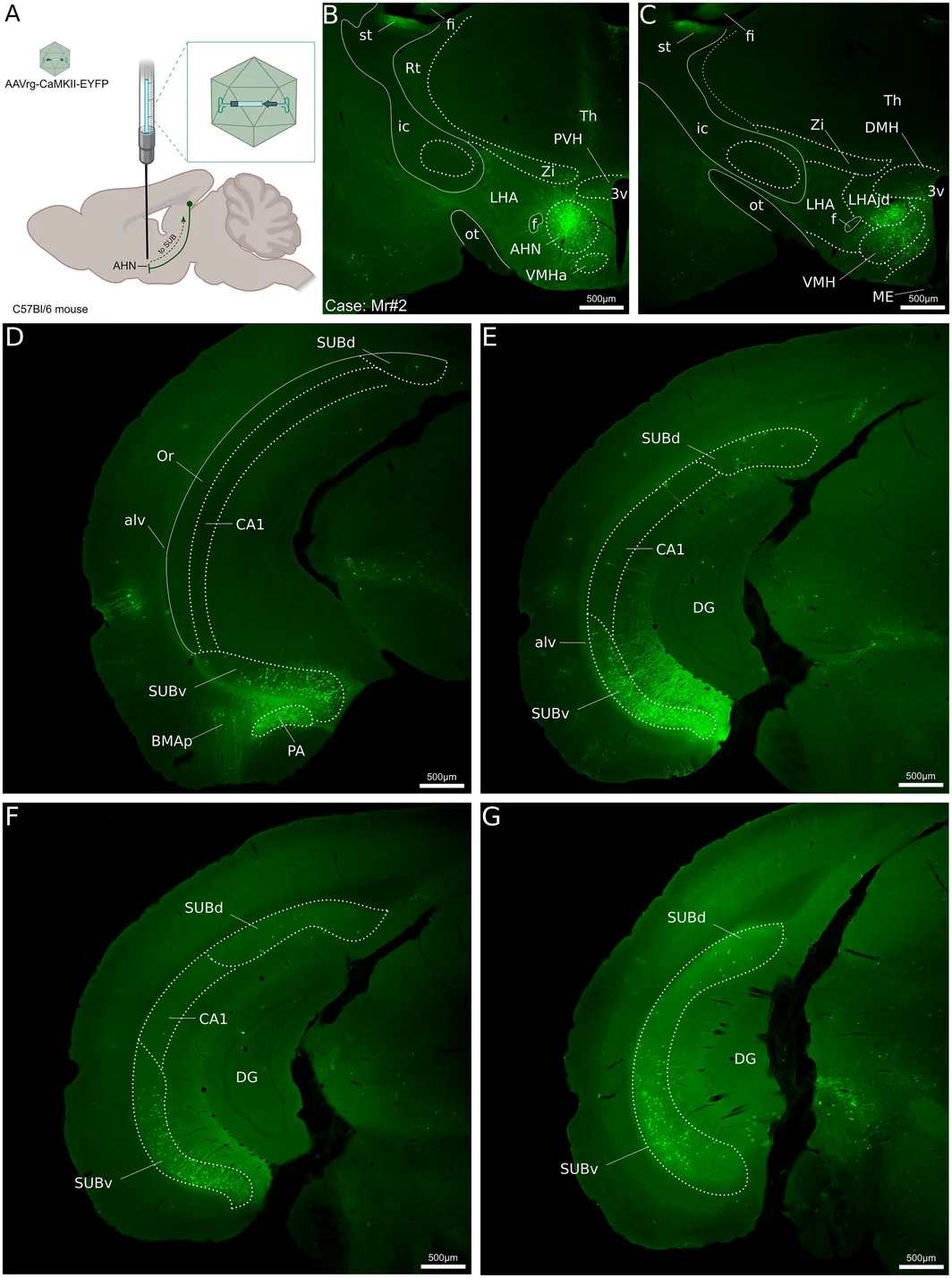
Distribution of hippocampal cells projecting to the anterior hypothalamic region. (A) Schematic representation of the retrograde viral tracing experimental design. (B–C) Injection site of a representative case (experiment MR#2) illustrating the AAVretro‐CaMKII‐EYFP injection centered in the anterior hypothalamic nucleus (B) spreading to the juxtadorsomedial region of the lateral hypothalamic area and ventromedial nucleus (C). (D–G) Experiment MR#2—Fluorescent photomicrographs of serial frontal sections illustrating the distribution of EYFP retrogradely labeled cells across the rostro‐caudal extent of the hippocampal region. For abbreviations see list.

We investigated the distribution of hippocampal cells that directly project to the anterior hypothalamic region, using an AAV retro vector carrying the enhanced yellow fluorescent protein (EYFP) reporter gene (Figure 1A). In three experiments, we obtained injection sites centered in the anterior hypothalamic region. For our results, we focused on experiment MR#2, which showcased the most extensive labeling in the hippocampal region (Figure 1). In this experiment, the injection site was centered in the anterior hypothalamic nucleus and extended to a certain degree to adjacent areas, including the lateral hypothalamic juxtadorsomedial region and the ventromedial hypothalamic nucleus (Figure 1B,C). As illustrated in Figure 1, retrogradely labeled cells were primarily pyramidal neurons distributed throughout the pyramidal layer of the ventral subiculum (Figure 1D–G). Additionally, a small group of labeled cells was identified in the pyramidal layer of the dorsal subiculum (Figure 1E–G). Importantly, in all instances, we observed only scattered labeled cells in the adjacent CA1 field (Figure 1D–F), confirming that the ventral subiculum contains the majority of hippocampal cells projecting to the anterior hypothalamic region. In three female mice, we assessed the effectiveness of the AAVrg‐hSyn‐Cre‐WPRE‐hGH injection in the anterior hypothalamic region for providing Cre‐mediated insertion in subicular cells using Ai6 female mice (for schematics see Figure S1A). This useful Cre reporter strain expresses robust ZsGreen1 fluorescence following Cre‐mediated recombination. As shown in the Supporting Information material (Figure S1, experiment FE#06), we observed robust ZsGreen1 fluorescence following Cre‐mediated recombination throughout the subiculum, providing a more widespread view of the entire subicular fields projecting to the anterior hypothalamic region. Compared to experiments using the AAV retrovector carrying the EYFP reporter gene, we obtained a much larger population of labeled cells, providing greater sensitivity for detecting the entire subicular population projecting to the anterior hypothalamic region. As in experiment MR#2, the vast majority of labeled cells were found in the ventral SUB, with fewer cells in the dorsal SUB and only scattered cells in CA1 field (see Figure S1).

### 3.2 Collateral Branching Pattern of Subicular Cells Projecting to the Anterior Hypothalamic Region

**FIGURE 2:**
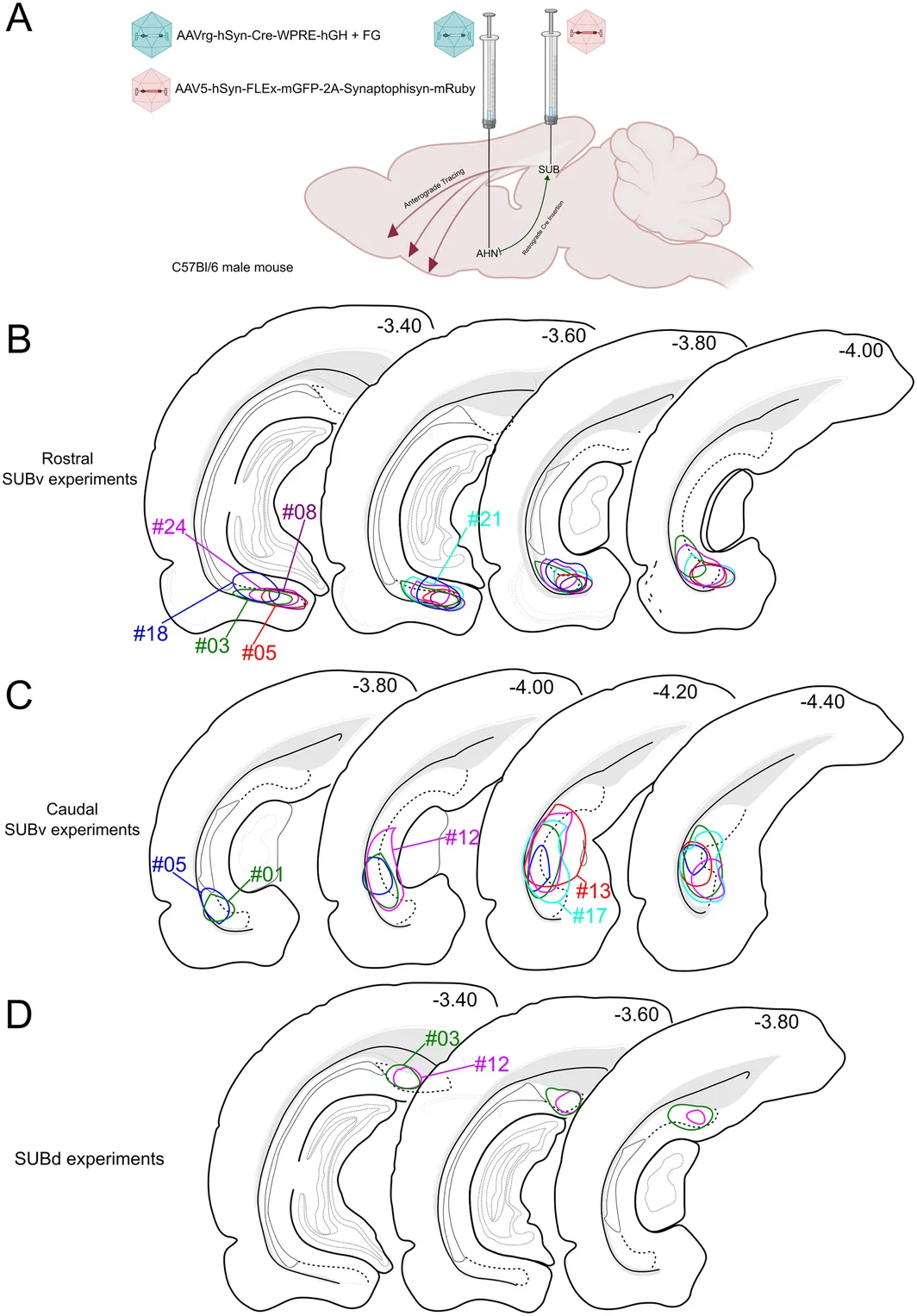
Experimental investigation of the projections from the SUB. (A) Schematic representation of the intersectional approach of experimental design. (B–D) Schematic representation showing the distribution and extent of the injection sites located in the rostral two‐thirds (B) and caudal half (C) of the ventral subiculum, and dorsal subiculum (D). Numbers on the top right of each section depict the approximate distance from the bregma. For abbreviations see list.

To investigate the collateral branching pattern of subicular neurons that project to the anterior hypothalamic region (AH), we performed a double injection procedure. First, we injected an AAV retro vector into the anterior hypothalamic region to insert Cre recombinase specifically in the subicular neurons projecting to the AH. Second, into the subiculum, we injected an AAV vector expressing membrane‐bound GFP and the presynaptic protein synaptophysin fused to the red fluorescent protein mRuby in a Cre‐dependent manner (for schematics, see Figure 2A). Importantly, the control cases that received only ventral subicular injections of a Cre‐dependent AAV vector expressing membrane‐bound GFP and the red fluorescent protein mRuby, but that did not receive the AAV retro vector into the anterior hypothalamic area to insert Cre recombinase into ventral SUB‐projecting cells, did not label any subicular cells. This approach allowed us to label the entire branching projection pattern of subicular neurons that project to the AH.

In males, anterograde tract tracing using an AAV vector labeled cells confined to the ventral subiculum (ventral SUB) in 11 cases, while in the other two cases, it labeled cells in the dorsal subiculum (dorsal SUB). For the ventral SUB, six of the injections labeled cells located in the rostral two‐thirds (Figure 2B), while five cases presented cell labeling in the caudal half (Figure 2C). Moreover, we obtained three injections in the ventral SUB of females, two located in the rostral two‐thirds and another spreading to the caudal third of the ventral SUB (Figure S2B).

#### 3.2.1 Projections From the Rostral Two‐Thirds of Ventral SUB

**FIGURE 3:**
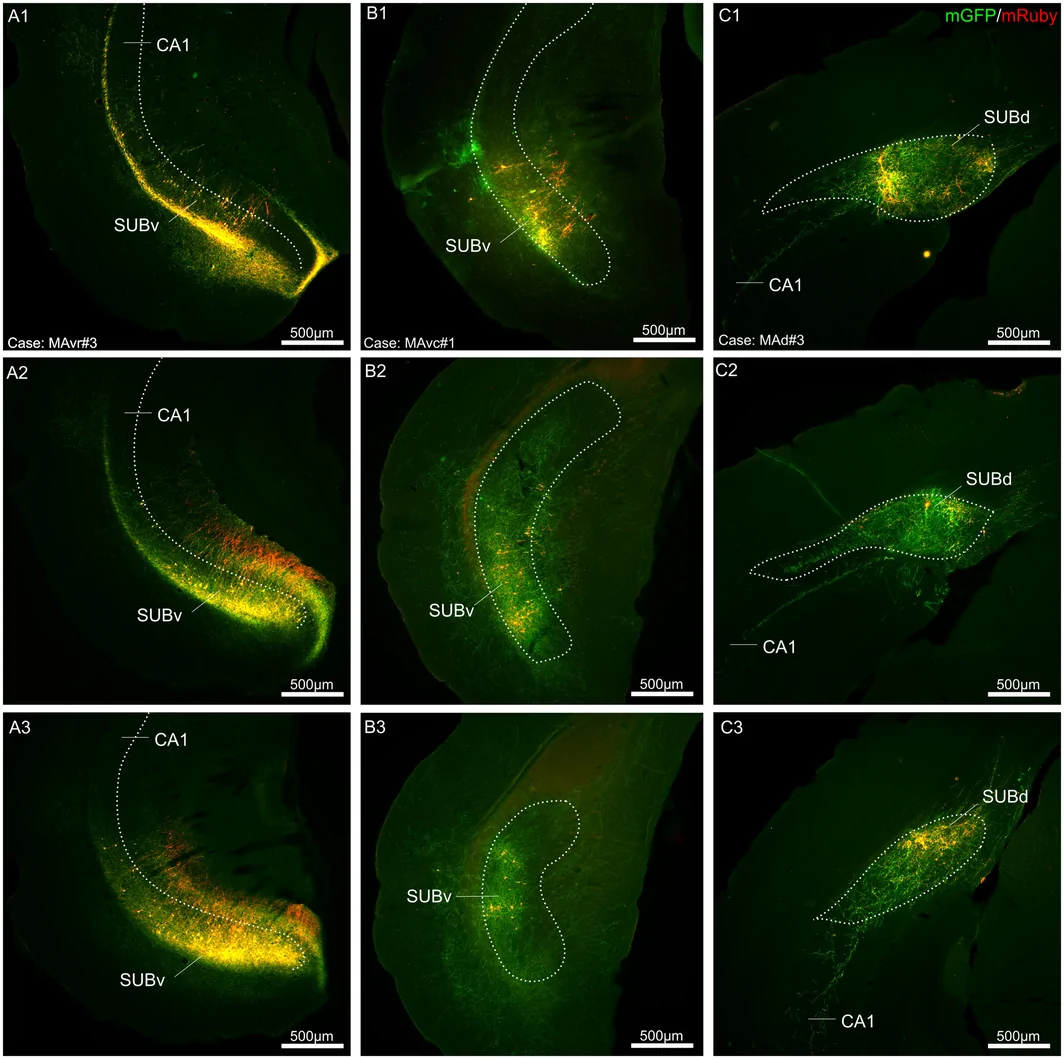
Experimental investigation of the projections from the SUB. Fluorescent photomicrographs of frontal section series showing the rostro‐caudal distribution of the injection sites (GFP and mRuby labeled cells) of representative injections in the rostral two‐thirds of the ventral SUB—experiment MAvr#3 (A1–A3), the caudal half of the ventral SUB—experiment MAvc#1 (B1–B3), and the dorsal subiculum—experiment MAd#3 (C1–C3).

In eight experiments, six in males and two in females, the injection sites were located in the rostral two‐thirds of the ventral SUB. Overall, all experiments presented a similar projection pattern, with no clear differences between males and females. To illustrate in detail the distribution of branching projections from the rostral ventral SUB providing projections to the AH, we chose experiment MAvr#3 because it presented an injection site with a larger number of labeled cells extending along the rostral two‐thirds of the ventral subiculum (Figure 3A1–A3). Putative differences from other cases will be noted. For the sake of completeness, in Supporting Information material, we illustrated a representative experiment in a female with an injection centered in the rostral half of the ventral SUB (experiment FE#6, Figure S3). Moreover, in the Supporting Information material, we illustrated experiment MAvr#24, with an injection centered in the rostral two‐thirds of the ventral SUB, showing a projection pattern similar to that of experiment MAvr#3, to compare with experiment MAc#17, which had an injection site in the caudal pole of the ventral SUB (see Figure S5).

##### 3.2.1.1 Projections to the Temporal Cortex and Amygdala

**FIGURE 4:**
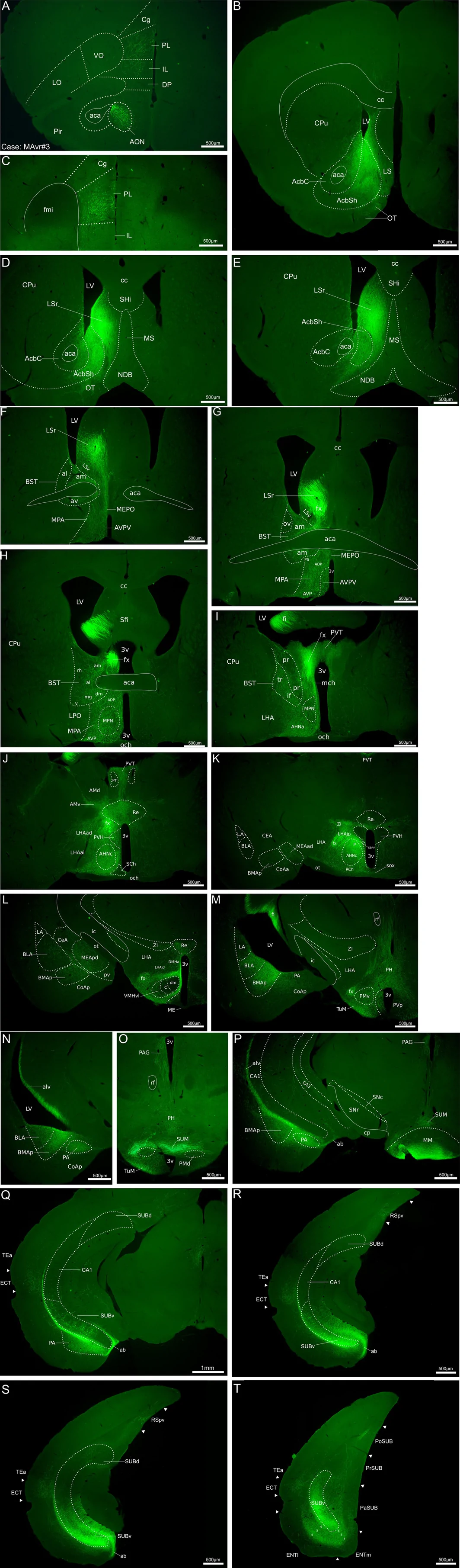
Projections from the rostral two‐thirds of the ventral SUB. (A–T) Fluorescent microphotographs showing a series of frontal sections arranged from rostral to caudal illustrating the distribution of GFP anterogradely labeled fibers in experiment MAvr#3, in which the injection site is illustrated in Figure 3A1–A3. For abbreviations see list.

In the hippocampus, rostral ventral subicular projecting to the AH targets the caudal parts of the ventral subiculum, where a dense terminal field was noticed (Figure 4T). One group of labeled fibers courses directly from the injection site to the temporal cortex and amygdala by crossing the angular bundle (Figure 4P–S). At caudal levels, these fibers form a dense terminal field aimed at layers 4–6 of the medial and lateral entorhinal cortices (Figure 4T). A small group of these fibers continues dorsolaterally and provides relatively sparse projections to the ectorhinal and temporal associative areas (Figure 4Q–T). In the amygdala, at caudal levels, particularly dense terminal fields were observed in the posterior nucleus and posterior basomedial nucleus, extending to the adjacent posterior basolateral nucleus (Figure 4M–Q). Proceeding rostrally, fibers provide a moderate projection field to the posterior basomedial and medial amygdalar nuclei (Figure 4K,L). Note that in the medial amygdalar nucleus, labeled fibers project particularly to the posterodorsal and anterodorsal parts (Figure 4K,L). At very rostral amygdalar levels, we also noted a sparse projection to the nucleus of the lateral olfactory tract.

##### 3.2.1.2 Projections Through the Fimbria

The second major group of labeled axons leaving the ventral subiculum courses through the alveus and fimbria and, on reaching the septum, divides into three components: the precommissural fornix, the postcommissural fornix, and the medial corticohypothalamic tract.

###### 3.2.1.2.1 Precommissural Fornix

As the fimbria approaches the caudal end of the septal region, axons comprising the precommissural fornix fan out to run through the septal region, projecting to the lateral septal nucleus and several other targets, including the anterior olfactory nucleus, the prefrontal cortex, the nucleus accumbens, the bed nuclei of the stria terminalis (BST), and the preoptic region (Figure 4A–I).

In the septal region, the ventral part of the rostral lateral septal nucleus receives a massive projection extending to a lesser degree to the ventral lateral septal nucleus, which contains a sparser number of labeled fibers (Figure 4D–G). At rostral levels of the septal region, a large contingent of fibers projects to the nucleus accumbens, where a very dense terminal field is found in the shell region of the nucleus, extending laterally to provide substantial input to the medial parts of the core region (Figure 4B,D,E). A small contingent of these fibers continues ventrally to project to the olfactory tubercle, where we found small clusters of terminal fields (Figure 4B,D). Proceeding rostrally, a contingent of fibers from the precommissural fornix continues to project to the anterior olfactory nucleus, and a relatively small number of fibers continues dorsally to project to the infralimbic, prelimbic, and anterior cingulate cortical fields (Figure 4A–C). At caudal levels of the septal region, fibers from the precommissural fornix take a ventral course to project to the anterior division of the BST or continue immediately rostral to the anterior commissure to project to the preoptic region and more caudal parts of the BST (Figure 4F–I). In BST, moderate projections were found in the anterior division of the BST aimed at the anteromedial, anterolateral, magnocellular, and dorsomedial areas (Figure 4F–H), while in the posterior division of the BST, projections were aimed mostly at the interfascicular and transverse nuclei and tended to avoid the principal nucleus (Figure 4I). In the preoptic region, an expressive terminal field covered the entire medial preoptic area, providing substantial projection to the median preoptic, anterodorsal preoptic, parastrial, anteroventral preoptic, anteroventral periventricular, and medial preoptic nuclei (Figure 4G–I).

###### 3.2.1.2.2 Medial Corticohypothalamic Tract and Postcommissural Fornix

The medial corticohypothalamic tract contained a substantial number of labeled fibers seen to diverge from the postcommissural fornix in the anterodorsomedial tip of the hypothalamus, between the paraventricular nucleus of the thalamus and hypothalamus (Figure 4I). In addition, in our experiments, a massive number of labeled fibers traveled through the postcommissural fornix, giving rise to the descending column of the fornix (Figure 4I–M). Fibers coursing through these pathways provide extensive projections to the thalamus and hypothalamus.

In the thalamus, the rostral pole of the paraventricular nucleus and nucleus reuniens presented massive projections (Figure 4I,J), which also extended laterally to provide significant inputs to the ventral part of the anteromedial thalamic nucleus (Figure 4J). Interestingly, proceeding caudally through the thalamus, the nucleus reuniens and the paraventricular nucleus contained fewer labeled fibers (Figure 4K,L). In the hypothalamus, rostrally at anterior hypothalamic levels, an extensive terminal field covered the anterior part of the anterior hypothalamic nucleus (Figure 4I). Proceeding caudally in the anterior hypothalamic area, dense projections covered the entire medial hypothalamic zone, including the perisuprachiasmatic area, retrochiasmatic area, subparaventricular region, and anterior hypothalamic nucleus, extending laterally to the adjacent lateral hypothalamic fields, to include the anterodorsal and juxtaparaventricular areas (Figure 4J,K). Importantly, at these levels, a particularly dense terminal field was distributed to the subparaventricular zone and posterior and central parts of the anterior hypothalamic nucleus, while the labeled fibers tended to avoid the paraventricular nucleus and the periventricular region (Figure 4K). At tuberal levels, the medial zone presented a robust projection aimed at the cell‐sparse “shell” surrounding the dorsomedial and central parts of the ventromedial nucleus, the core region of the ventrolateral part of the ventromedial nucleus, and the tuberal nucleus, in addition to sparser projections to the dorsomedial nucleus (Figure 4L). In the mammillary region, at pre‐mammillary levels, anterograde‐labeled fibers provide projections that cover the ventral premammillary nucleus, the posterior periventricular nucleus, and the tuberomammillary nucleus, and tend to avoid the dorsal premammillary nucleus (Figure 4M,O). These fibers continue dorsally to the posterior hypothalamic nucleus, which contains a significant projection field (Figure 4M). At mammillary levels, massive bilateral projections target the supramammillary nucleus and the ventral part of the medial mammillary nucleus (Figure 4O,P). From the mammillary region, only a sparse number of fibers ascend through the periventricular region on the way to project to the periaqueductal gray (Figure 4O).

#### 3.2.2 Projections From the Caudal Half of Ventral SUB

In a series of six experiments involving five males and one female, the injection sites included the caudal ventral SUB. Among the males, five injection sites were located in the caudal half, with three of these injections centered in the caudal third of the ventral SUB (Figure 2C). In females, in one experiment the injection site extended to the caudal half of the ventral SUB (Figure S2B).

**FIGURE 5:**
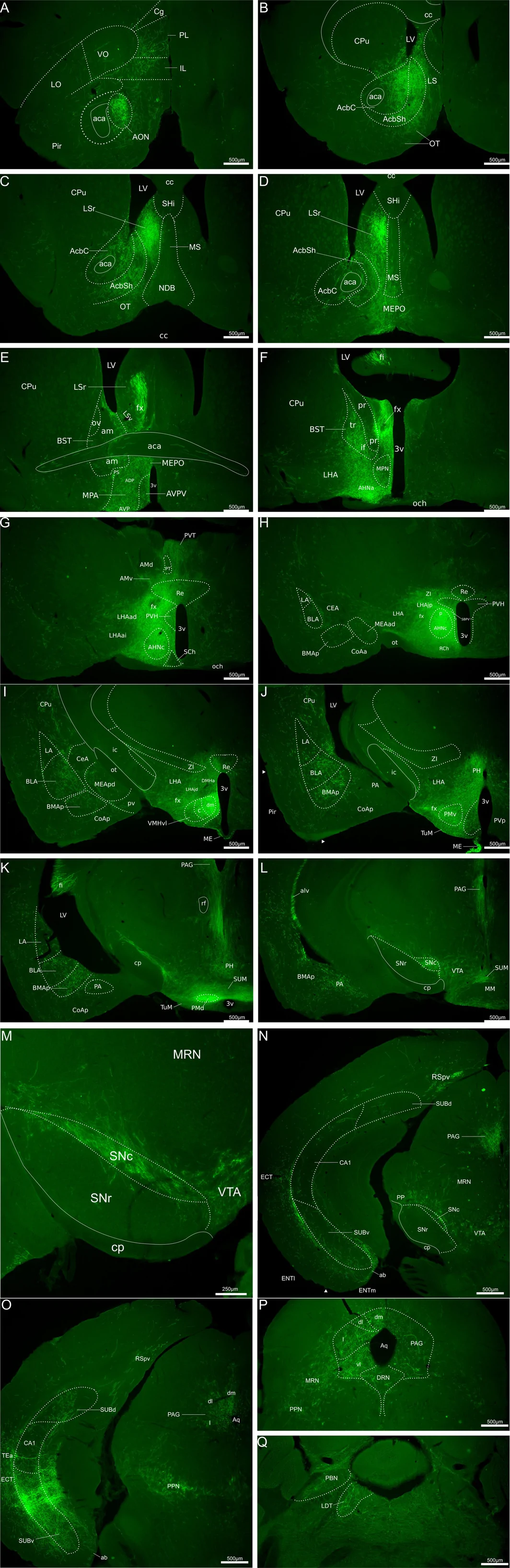
Projections from the caudal half of ventral SUB. (A–Q) Fluorescent microphotographs showing a series of frontal sections arranged from rostral to caudal illustrating the distribution of GFP anterogradely labeled fibers in the experiment MAvc#1, which the injection site is illustrated in Figure 3B1–B3. For abbreviations see list.

To illustrate in detail the distribution of branching projections from the caudal ventral SUB providing projections to the AH, we chose experiment MAvc #1 (Figure 3B1–B3), because the injection site extends across the caudal half of the ventral SUB (Figure 2C) and the case presents a clear definition of the projection fields (Figure 5). Putative differences from other experiments with injection sites more restricted to the caudal third of the ventral SUB will be noted (Figure S5). For the sake of completeness, in Supporting Information material, we illustrate an experiment in a female with injection centered in the caudal half of the ventral SUB (Experiment FE#3; Figure S4).

In the hippocampus, contrasting with the rostral cases, projection fibers hit the dorsal subiculum, and a few of them continue dorsally to project to the ventral retrosplenial area (Figure 5N,O). The projections to the temporal cortex and amygdala were far weaker and more widespread than those previously described for rostral cases. Compared to rostral cases, a sparser group of labeled fibers leaves the hippocampus through the angular bundle and projects to the entorhinal cortex and amygdala (Figure 5H–L,N,O). In the temporal cortex, we observed projections to the medial and lateral entorhinal cortices, which extend dorsally to project to the ectorhinal and temporal associative cortices (Figure 5N,O). In the amygdala, as for the rostral cases, the primary targets are the posterior and the posterior basomedial nuclei, as well as the posterior basolateral nucleus (Figure 5I–L). Notably, caudal ventral SUB projecting fibers to the amygdala may extend dorsally to the caudoputamen (Figure 5I,J). Moreover, at amygdalar levels, a small contingent of caudal ventral SUB projections extends laterally to project to the piriform cortex (Figure 5J–L). Interestingly, for injections restricted to the caudal third of the ventral SUB, the projection to the amygdala targets the basolateral nucleus, with no projection to other targets such as the posterior and posterior basomedial nuclei, and fibers projecting to the amygdala extend dorsally to provide a denser projection to the caudoputamen. (Figure S5N,P,R).

The majority of fibers originating from the caudal ventral SUB travel through the alveus and fimbria. At the septal level, these fibers divide into the pre‐commissural and post‐commissural fornix. The post‐commissural fibers from the caudal ventral SUB enter the descending column of the fornix (Figure 5F,J).

Fibers from the caudal ventral SUB that pass through the precommissural fornix are positioned more medially (Figure 5E) and project to several areas, including the prefrontal cortex, the nucleus accumbens, the lateral septal nucleus, the bed nucleus of the stria terminalis (BST), and the preoptic region. Within the septal area, the caudal ventral SUB particularly targets the rostral lateral septal nucleus, creating a notable projection that is more medially positioned than those originating from the rostral ventral SUB (Figures 5C,D and S5F). In the nucleus accumbens, the caudal ventral SUB has a dense projection to the shell and adjacent parts of the core region (Figure 5B–D). In comparison to the projections from the rostral ventral SUB, the caudal ventral SUB projects more heavily to the rostral parts than to the caudal parts of the nucleus accumbens (Figure 5B–D). This difference is even more striking for injections restricted to the caudal third of the ventral SUB, where the projection to nucleus accumbens is restricted to its rostral parts (Figure S5C–F). A group of fibers continues rostrally to project to the anterior olfactory nucleus and extends dorsally to provide inputs to the infralimbic and prelimbic cortex (Figure 5A). At the level of the septal region, fibers from the precommissural fornix project to the BST and the preoptic region. In the BST, the projections to the anterior division are more limited compared to those from the rostral ventral SUB and target primarily the anteromedial area (Figure 5E). In contrast, the caudal division of the BST shows a correspondingly dense projection to the interfascicular and transverse nuclei while largely avoiding the principal nucleus (Figure 5F). Similar to the rostral ventral SUB projections, the caudal SUB provides extensive projection to the preoptic region covering the entire medial preoptic region and extending to a lesser degree to the lateral preoptic region (Figure 5D–F). In sharp contrast, for injections restricted to the caudal third of the ventral SUB, there is almost no projection to the BST and preoptic region (Figure S5G–L).

The majority of fibers from the postcommissural fornix enter the descending column of the fornix. These fibers make extensive projections to the thalamus, hypothalamus, and various sites in the brainstem. Within the thalamus, similar to the fibers originating from the rostral ventral SUB, the caudal ventral SUB projects extensively to the nucleus reuniens and the paraventricular nucleus and extends laterally to the ventral part of the anteromedial nucleus (Figure 5G). This projection is particularly concentrated at the rostral levels and decreases significantly in density at more caudal positions. (Figure 5G–I). Notably, for injections restricted to the caudal third of the ventral SUB, the projections to the thalamus are largely decreased (Figure S5K,L).

In the hypothalamus, the projections from the caudal half of the ventral SUB show a strikingly complementary pattern of innervation compared to those identified in the rostral two‐thirds of the ventral SUB. At the anterior levels of the hypothalamus, the caudal ventral SUB exhibits a similar projection pattern within the medial zone. However, it has a significantly greater number of fibers that project to the periventricular and lateral hypothalamic zones (Figure 5F–I). In the periventricular zone, a notable number of fibers target the paraventricular and periventricular nuclei, reaching areas containing neuroendocrine motor neurons (Figure 5H). Additionally, a substantial contingent of labeled fibers extends into the lateral zone, covering a broad area of the lateral hypothalamus (Figure 5F–H). Some of these fibers continue laterally through the ansa peduncularis to reach the amygdala (Figure 5H). At the tuberal levels, compared to the rostral cases, the dorsomedial nucleus receives heavier inputs from the caudal ventral SUB (Figure 5I). In addition, in contrast to the rostral ventral SUB, the caudal ventral SUB exhibits a prominent projection to the core region of the dorsomedial and central parts of the ventromedial nucleus, as well as a somewhat sparser projection to the ventrolateral part (Figure 5I). Likewise, injections restricted to the caudal third of the ventral SUB also provide a dense projection to the core region of the dorsomedial and central parts of the ventromedial nucleus (Figure S5P), which is also targeted in experiments with injections extending to the caudal ventral SUB in females (Figure S4D). Additionally, while a few labeled fibers extend to the arcuate nucleus, a clear network of labeled fibers was observed in the external lamina of the median eminence, which is known to contain nerve fibers that terminate on the primary plexus of the hypophysial portal vessels (Figure 5I). A similar projection to the external lamina of median eminence was confirmed in a female with injection site extending to the caudal half of the ventral SUB (Figure S4D). At the tuberal levels, the caudal ventral SUB shows a clear projection to the lateral hypothalamus (Figure 5I). At mammillary levels, compared to the rostral three‐quarters of the ventral SUB, the caudal ventral SUB also provides similarly strong projections to the tuberomammillary, ventral premammillary, and supramammillary nuclei (Figure 5I,J), while in contrast, it provides bilateral dense terminal fields to the dorsal premammillary nucleus (Figure 5K), which is also particularly targeted in the experiment with injection in the caudal half of the ventral SUB in females (Figure S4E). A large contingent of fibers from the caudal ventral SUB continue dorsally to the posterior hypothalamic nucleus, providing a significant projection field (Figures 5I,J and S5R,T).

Importantly, in sharp contrast to what was found for injections in the rostral two‐thirds of the ventral SUB, a large contingent of fibers from the caudal ventral SUB provides significant projections to several sites in the brainstem. We also found that the same projection pattern to the brainstem in experiments with injections in the caudal third of the ventral SUB and in the injection placed in the caudal half of the ventral SUB in females (Figure S4F–H). Thus, from the mammillary region, fibers may continue caudally to project to the ventral tegmental area and pars compacta of the substantia nigra or take a dorsal course through the periventricular region to enter the periaqueductal gray (Figure 5L). The ventral tegmental area receives a significant plexus of labeled fibers that extends dorsolaterally to project to the compact part of the substantia nigra, and a few of these fibers extend laterally and reach the peripeduncular area (Figures 5L–N and S4F). Fibers coursing through the dorsal pathway project to the periaqueductal gray, where, at rostral levels, they provide a projection to the dorsal and lateral parts of the periaqueductal gray (Figures 5O and S4G). As they continue more caudally, they form a clear terminal field in the dorsal, lateral, and ventrolateral parts of the periaqueductal gray (Figure 5N–P). A contingent of fibers coursing through the periaqueductal gray extends ventrolaterally to project to the mesencephalic reticular and pedunculopontine nuclei, which contain a clear terminal field (Figures 5O,P and S4G). A contingent of fibers coursing through the periaqueductal gray continues caudally to project to the laterodorsal tegmental nucleus, and to a lesser degree to the parabrachial region (Figures 5Q and S4H).

#### 3.2.3 Projections From the Dorsal SUB

To illustrate the distribution of branching projections from the dorsal SUB cells that receive Cre insertion from an AAV retrograde vector placed in the anterior hypothalamic region, we chose experiment MAd#3 because it contained a significant number of labeled cells in the dorsal subiculum (Figure 3C1–C3,C5).

**FIGURE 6:**
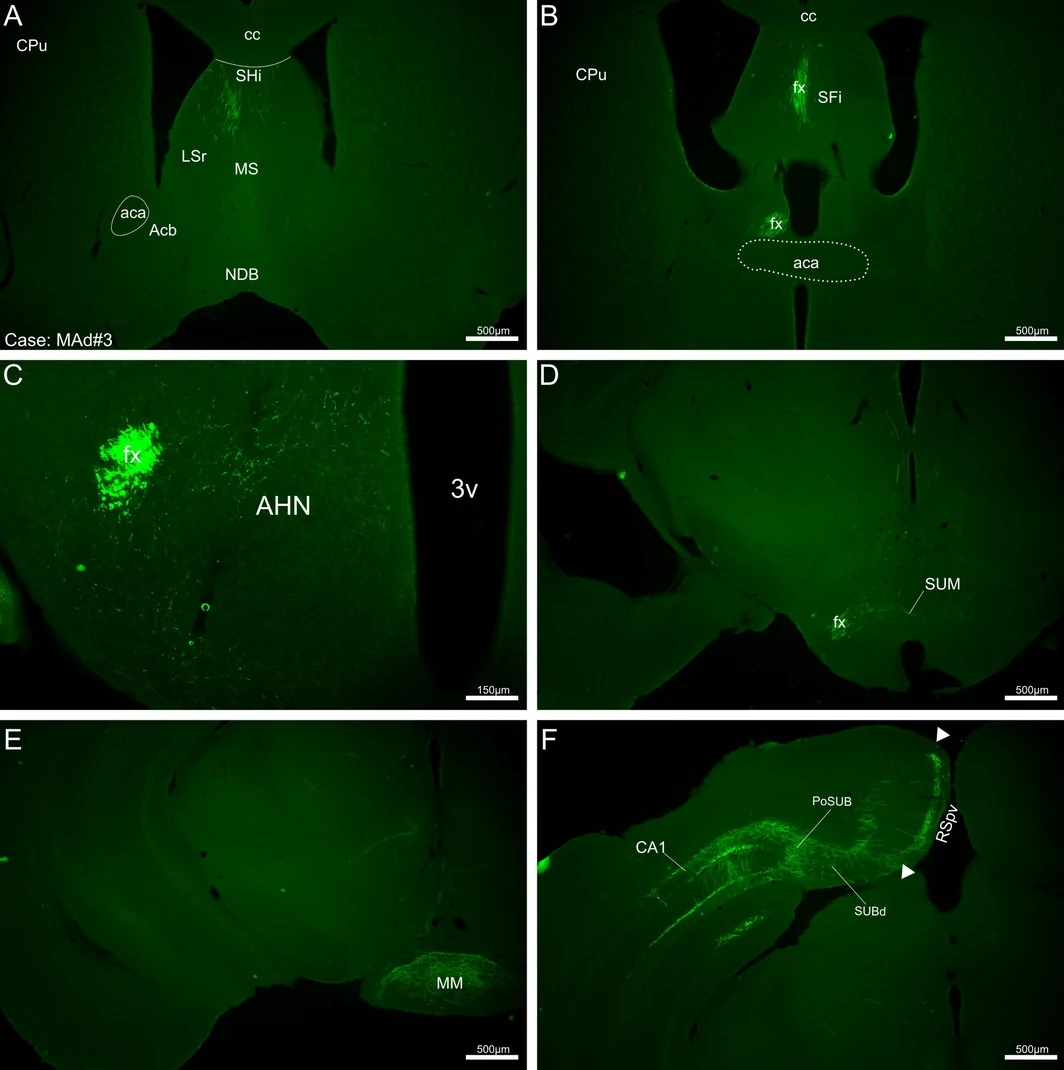
Projections from the dorsal SUB. (A–F) Fluorescent microphotographs showing a series of frontal sections arranged from rostral to caudal illustrating the distribution of GFP anterogradely labeled fibers in the experiment MAd#3, which the injection site is illustrated in Figure 3C1–C3. For abbreviations see list.

Compared to the ventral SUB, the dorsal SUB cells projecting to the anterior hypothalamic region exhibit a somewhat limited branching pattern. From the injection site, labeled fibers extend medially to provide a dense terminal field in the superficial layers of the retrosplenial cortex (Figure 6F). The second major group of labeled axons that leave the dorsal subiculum courses through the alveus and fimbria (Figure 6C) and, on reaching the septum, divides into the pre‐ and postcommissural fornix (Figure 6A,B). The precommissural fornix enters the septal region, providing a relatively sparse projection to the medial parts of the rostral lateral septal nucleus (Figure 6A). Fibers from the postcommissural fornix continue caudally through the descending columns of the fornix. Notably, only scattered fibers leave the fornix to project to adjacent hypothalamic regions, including very sparse projections to the anterior hypothalamic area (Figure 6C). By and large, these fibers continue to the mammillary region, projecting to the supramammillary nucleus and the dorsal part of the medial mammillary nucleus, which contains a particularly dense terminal field (Figure 6D,E).

## 4 Discussion

This investigation reveals that hippocampal cells providing direct inputs to the anterior hypothalamic area exhibit extensive branching patterns, extending to multiple brain regions. Given the central role of this pathway in mediating fear and anxiety, our findings suggest that the ventral hippocampal regions involved in processing these responses may exert a significant influence over various brain areas. Consequently, this influence likely impacts diverse functions, including memory, motivation, arousal, and circadian rhythms, as well as the regulation of neuroendocrine processes and goal‐oriented behaviors.

Using an AAV retro vector carrying EYFP, we first identified the location and extent of hippocampal cells that project to the AH. Our results showed that these hippocampal cells are largely distributed along the rostro‐caudal axis of the ventral subiculum. Next, by injecting AAV retro vectors into the AH to introduce Cre recombinase into the hippocampal projecting cells, and by administering AAV vectors that express GFP and mRuby‐labeled synaptophysin in a Cre‐dependent manner into the ventral subiculum, we visualized the complete projection patterns of the subicular cells providing direct inputs to the AH. Our results showed that ventral subicular anterior hypothalamic‐projecting cells give rise to a projection pattern similar to that previously described for the ventral subiculum using the *Phaseolus vulgaris* leucoagglutinin (PHA‐L) tracing method (Canteras and Swanson 1992). Interestingly, compared with previous PHA‐L studies, the present study provides a more complete view of the projection fields because viral tracing tools are more sensitive and are likely to reveal them in their entirety. Moreover, injections placed in the caudal half of the ventral subiculum reveal a pattern complementing that seen in rostral cases, showing dense projection to the core region of the dorsomedial and central parts of the ventromedial nucleus, significant projections to the neuroendocrine‐related areas, including the paraventricular hypothalamic nucleus and external lamina of median eminence, and clear inputs to brainstem sites, such as the ventral tegmental area, pars compacta of substantia nigra, periaqueductal gray, and pedunculopontine nucleus.

Given the novelty of our findings regarding the unusual projections of the caudal ventral SUB, it is crucial to ascertain whether these projections originate from sources other than subicular cells. Notably, in these cases, transfected cells expressing Cre‐dependent membrane‐bound green fluorescent protein (GFP) and the presynaptic protein synaptophysin fused to the red fluorescent protein mRuby were predominantly observed in subicular neurons, with only occasional labeling of neurons in the dentate gyrus, which are known only to provide intrinsic projections to the hippocampus. Alternatively, one might argue that AAV5 expressing membrane‐bound GFP and mRuby in a Cre‐dependent manner, injected into the ventral SUB, could retrogradely transfect Cre‐positive neurons in the AH. This could potentially explain the brainstem projections attributed to the caudal ventral SUB. However, as previously mentioned, cells expressing Cre‐dependent membrane‐bound GFP and synaptophysin fused to mRuby were largely restricted to subicular neurons, with no labeled cells found in the AH. Consequently, AH cells did not contribute to the anterograde labeling observed in our experiments. If this were the case, we would also expect to see similar results (i.e., projections to the unusual targets) from injections into the rostral ventral SUB. Furthermore, retrograde transport from ventral SUB injections to the AH is highly unlikely, as the AH has only sparse projections to the ventral SUB (Risold et al. 1994). Additionally, it is important to note that most of the unusual subicular targets reported in our study, specifically, the external lamina of the median eminence, the ventral tegmental area, the substantia nigra, and the pedunculopontine nucleus, are not or are only minimally targeted by the AHN (Risold et al. 1994). Taken together, the evidence strongly supports that our anterograde tract tracing results are convincing and reveal these unusual projections from the caudal ventral SUB.

In the cerebral cortex, ventral subicular anterior hypothalamic projecting cells provide strong inputs to the lateral and medial entorhinal areas, as well as sparser projections to the ectorhinal and adjacent temporal associative regions, as well as to the prelimbic and anterior cingulate cortices. In the entorhinal areas, projection preferentially targets layers 4–6, which contain dense terminal fields. Here, it is noteworthy that deep layers of the lateral entorhinal area project to the entire cortical mantle, mainly to the superficial layers (Swanson and Kohler 1986), with potential roles in influencing arousal. In addition, the lateral entorhinal area provides dense projections to the lateral and, to a lesser degree, the anterior basolateral amygdalar nuclei, as well as to the medial parts of the nucleus accumbens (Swanson and Kohler 1986), representing alternative ways for the ventral subiculum to influence these brain sites. The medial prefrontal cortex, particularly the prelimbic and anterior cingulate areas, receives a relatively sparse projection from cells in the ventral subiculum that target the AH. Here, it is important to highlight that the ventral hippocampal–medial prefrontal cortical pathway has been significantly associated with anxiety responses. Research has demonstrated that ventral hippocampal neurons projecting to the prefrontal cortex exhibit anxiety‐related firing patterns (Ciocchi et al. 2015). Furthermore, studies have identified a specific role for the ventral hippocampal–medial prefrontal cortex projection in anxiety‐related behavior and the spatial representation of aversive information within the medial prefrontal cortex (Adhikari et al. 2010; Padilla‐Coreano et al. 2016). Additionally, ventral hippocampal projections to the prefrontal cortex have been linked to emotional, social, and object‐based memory (Bakoyiannis et al. 2023).

We have demonstrated that ventral subicular cells projecting to the AH extensively target the amygdala. Notably, there are particularly dense terminal fields in the posterior basomedial and posterior amygdalar nuclei, as well as clear inputs to the posterior basolateral nucleus, the anterodorsal and posterodorsal regions of the medial nucleus. Research indicates that the ventral hippocampus projections to the basal amygdala are implicated in emotional memory (Bakoyiannis et al. 2023). Additionally, the amygdalar targets of ventral subicular cells projecting to the AH may represent critical alternative pathways to both the nucleus accumbens and the medial hypothalamic zone. The posterior basomedial amygdalar nucleus has dense projections to the shell region of the nucleus accumbens and to the core region of the dorsomedial part of the ventromedial hypothalamic nucleus (Petrovich et al. 1996). Conversely, the posterior nucleus, along with the posterodorsal and anteroventral medial amygdalar nuclei, provides significant projections to various components of the reproductive circuit within the medial zone of the hypothalamus (Canteras et al. 1992, 1995). This circuit includes the medial preoptic nucleus, tuberal nucleus, ventrolateral part of the ventromedial nucleus, and ventral premammillary nucleus (Gross and Canteras 2012).

The nucleus accumbens is a key target of ventral subicular cells that project to the AH. These cells provide a significant direct projection that primarily targets the shell region of the nucleus accumbens and extends laterally to the core region. Additionally, this projection may be enhanced through indirect pathways involving the anterior paraventricular thalamic nucleus, the posterior basomedial amygdalar nucleus, the entorhinal cortex, and the ventral tegmental area, all of which provide particularly dense projections to the nucleus accumbens (Engelke et al. 2021; Petrovich et al. 1996; Swanson and Kohler 1986).

In rodents, stimulation of the ventral hippocampus, but not the dorsal hippocampus, increases locomotion by engaging the nucleus accumbens (Peleg‐Raibstein and Feldon 2006; Zhang et al. 2002). Conversely, inhibiting the ventral hippocampus leads to a decrease in locomotion (Bast et al. 2001). This connection with the nucleus accumbens plays an important role in reward‐directed and goal‐oriented functions. Notably, goal‐directed activity was evident in ventral hippocampal neurons that project to the nucleus accumbens (Ciocchi et al. 2015), and chemogenetic activation of these neurons enhances motivation to obtain palatable food (Singh et al. 2023). Additionally, pyramidal cells in the ventral hippocampus that project to the nucleus accumbens play a crucial role in storing social memories and significantly influence decisions to approach new social situations (Okuyama et al. 2016). Furthermore, this circuit is involved in regulating susceptibility to chronic social defeat (Bagot et al. 2015). Given the extensive projection of anterior hypothalamic ventral SUB‐projecting cells to the nucleus accumbens (NAc), it is important to study how these large branches of a pathway, crucial for mediating fear and anxiety, affect NAc function in reward‐directed and goal‐oriented functions and social interactions.

In the septal region, ventral subicular cells that project to the AH form a particularly dense connection to the rostral lateral septal nucleus (LS) and provide significant inputs to both the anterior and posterior divisions of the BST. The ventral hippocampal–LS pathway is known to influence anxiogenic behavior (LeDoux 2000). Pharmacological blockade of this pathway has anxiolytic effects, as it increases open‐arm exploration in the elevated plus maze (Trent and Menard 2010). Additionally, optogenetic activation of ventral hippocampal projections in the LS has been shown to reduce food intake (Sweeney and Yang 2015). Conversely, activation of the ventral SUB–BST pathway has been found to decrease contextual fear expression (Kopaeva et al. 2023). This aligns with findings suggesting that the ventral SUB–BST pathway is more active in neutral, familiar contexts compared to aversive ones (Urien et al. 2022). Therefore, it appears that the ventral SUB–BST pathway plays a role in behavioral responses to nonaversive contexts. Furthermore, selective activation of ventral SUB neurons projecting to the anterior BST has been shown to enhance the hypothalamic–pituitary–adrenal axis response during an open‐field test (Marsh et al. 2025), indicating that the anterior BST may mediate neuroendocrine control.

In the thalamus, particularly dense projections were observed to the anterior paraventricular nucleus, nucleus reuniens, and ventral part of the anteromedial nucleus. The anterior paraventricular thalamic nucleus (PVT) is another important node through which neurons from the ventral subiculum that project to the AH influence the nucleus accumbens. Interestingly, distinct subtypes of neurons that project from the anterior PVT to the nucleus accumbens may modulate food‐seeking responses in opposing ways. One subpopulation of neurons that express the glucose transporter Glut2 and project to the nucleus accumbens tends to increase food‐seeking behavior (Labouèbe et al. 2016). In contrast, another group of neurons that express the enzyme glucokinase or the neuropeptide corticotropin‐releasing factor suppresses food‐seeking responses (Engelke et al. 2021; Kessler et al. 2021). Research in rodents has demonstrated that anterior PVT neurons play a critical role in choosing between opposing motivational behaviors, such as approach versus avoidance responses in conflict situations (Engelke et al. 2021). Of particular relevance, studies have shown that the anterior PVT may integrate food signals with information about potential predators, regulating approach versus avoidance responses during conflicts, such as foraging while at risk of a predatory attack (Engelke et al. 2021). In this context, it would be valuable to explore how the population of ventral subicular cells projecting to the AH, which mediate fear and anxiety responses, impacts the anterior PVT's role in selecting appropriate behaviors.

The nucleus reuniens is an important thalamic target for ventral subicular cells that project to the AH. It serves as a crucial link between the hippocampus and the prefrontal cortex, playing a significant role in behaviors that rely on their interactions (Vertes et al. 2015). The nucleus reuniens is essential for working memory performance, which depends on circuits connecting the hippocampus and prefrontal cortex (Vertes et al. 2015). Additionally, a neural circuit consisting of the medial prefrontal cortex, the nucleus reuniens, and the hippocampus regulates fear memory generalization (Xu and Südhof 2013). When the nucleus reuniens is silenced, fear generalization is enhanced, while activating nucleus reuniens' neurons decreases it (Xu and Südhof 2013). Given these findings, it would be intriguing to explore how ventral subicular cells that process fear and anxiety responses influence working memory and fear generalization through extensive inputs to the nucleus reuniens. We observed that the extensive projections to the nucleus reuniens extend laterally to cover the ventral part of the anteromedial thalamic nucleus (AMv). Importantly, the AMv is heavily targeted by the medial hypothalamic fear circuit (Gross and Canteras 2012) and, through its projections to the anterior cingulate area, plays a critical role in the acquisition of contextual fear memory in response to predatory threats (de Lima et al. 2022). Thus, the AMv may serve as an alternative pathway through which ventral subicular cells, likely involved in processing fear and anxiety, can influence fear memory.

Cells in the ventral subiculum that project to the AH provide a broad projection to the hypothalamus, covering the preoptic, anterior, tuberal, and mammillary regions. Considering the injections placed in the rostral three‐quarters of the ventral subiculum, the resulting projection pattern to the hypothalamus closely resembles what was previously described in earlier PAHL tract tracing studies (Canteras and Swanson 1992). In contrast, the caudal quarter of the ventral subiculum exhibited a complementary projection pattern that previous studies did not reveal. Notably, cells in the caudal ventral subiculum provide distinct inputs to the external lamina of the median eminence, larger projections to areas containing neuroendocrine motor cells, particularly the paraventricular nucleus, a dense terminal field in the core region of the dorsomedial and central parts of the ventromedial nucleus, bilateral dense inputs to the dorsal premammillary, and more extensive projections to the lateral hypothalamic area.

Previous studies suggested that the ventral hippocampus does not have direct projections to the neuroendocrine motor cells, particularly to the paraventricular nucleus. Instead, the influence of the ventral hippocampus on neuroendocrine control is thought to be mediated through various cells that project to the paraventricular nucleus, both within and outside the hypothalamus, such as the anterior BST, medial preoptic nucleus, and dorsomedial hypothalamic nucleus (Marsh et al. 2025; Simerly and Swanson 1988; Thompson et al. 1996). In contrast, the current findings indicate that caudal ventral subicular cells project directly to the paraventricular nucleus. Additionally, these cells provide direct inputs to the external lamina of the median eminence, where neurons release neurohormones into the hypophyseal portal blood vessels (Swanson 1987). This area serves as a crucial interface between the brain and the endocrine system. This observation is indeed unexpected. It would be interesting to investigate how this alleged glutamatergic input from the ventral hippocampus to the median eminence impacts the anterior pituitary cells, which are known to respond to glutamatergic transmission (Meeker et al. 1994; Villalobos et al. 1996).

One of the most striking observations from the present study is that ventral subicular cells provide dense projections that cover the extent of the medial hypothalamic zone, including the preoptic, anterior, tuberal, and premammillary regions. The hypothalamic medial nuclei form a column of very distinct cell groups comprising two partially distinct circuits critical for controlling defensive and reproductive behaviors (Swanson 2000). Thus, apart from heavy inputs to the elements of the medial hypothalamic defensive circuit, including the anterior hypothalamic nucleus, dorsomedial ventromedial hypothalamic nucleus, and dorsal premammillary nucleus, ventral subicular cells projecting to the anterior hypothalamic area also branch to project to the medial hypothalamic reproductive circuit, comprising the medial preoptic, tuberal, ventrolateral ventromedial, and ventral premammillary nucleus, all of which are critical for controlling reproductive responses, such as maternal behavior, sexual behavior, and social aggression (Gross and Canteras 2012; Swanson 2000). Moreover, in the medial hypothalamic area, ventral subicular‐projecting cells form a particularly dense terminal field extending over the subparaventricular region, a major target of the suprachiasmatic nucleus and a critical region for controlling circadian rhythmicity (Watts et al. 1987). In this regard, it is noteworthy that selective lesions of the medial corticohypothalamic tract, exclusively formed by fibers projecting from the ventral subiculum, disrupted the diurnal rhythm of plasma adrenal glucocorticoid levels (Fischette et al. 1980). Moreover, in the medial hypothalamic zone, we noted important projections to the tuberomammillary nucleus, an arousal‐associated cell group in the caudal hypothalamus (Sherin et al. 1998). Thus, in addition to influencing defensive responses, ventral subicular anterior hypothalamic‐projecting cells extend massively to other medial hypothalamic regions that control a wide range of reproductive behaviors, as well as circadian rhythmicity and arousal.

The ventral subicular cells may also extend laterally and project to the lateral hypothalamic area. In the present context, it is particularly relevant that ventral subicular cells projecting to the lateral hypothalamic area may target the orexinergic cell group. Notably, this cell group is involved in the maintenance of wakefulness, while through projections to the ventral tegmental area, it influences motivated, goal‐directed responses, including ingestive behaviors (Tsujino and Sakurai 2013). Therefore, the orexinergic cell group may serve as another target through which ventral subicular cells, which are involved in mediating fear and anxiety, can influence both wakefulness and motivation.

In the present study, we noted that injections placed in the caudal quarter of the ventral subiculum provide a hitherto undescribed subicular projection to distinct brainstem sites, including the ventral tegmental area, the pars compacta of the substantia nigra, the periaqueductal gray, and the pedunculopontine nucleus. The ventral tegmental area is required for a wide range of motivated behaviors (Stuber 2023). Interestingly, previous studies have shown a correlation between increased excitatory activity in the ventral hippocampus and ventral tegmental area, which may account for stress‐produced anxiety‐like responses, as well as impaired sociability and cognitive function in a rodent model of schizophrenia (Cavichioli et al. 2023). The periaqueductal gray, in turn, may account for another route by which ventral subicular cells influence motivated behaviors, particularly defensive and reproductive responses (Motta et al. 2017; Van der Horst and Holstege 1998). Clear projections were also noted to the pedunculopontine nucleus. The pedunculopontine nucleus is a cholinergic cell group that may serve as a site through which the ventral subiculum influences cortical arousal and behavioral locomotor activity (Benarroch 2013).

Previous studies that employed electrophysiological recording and conventional retrograde tract‐tracing methods revealed limited branching in subicular‐projecting cells (Ciocchi et al. 2015; Jimenez et al. 2018; Jin and Maren 2015; Urien et al. 2022). Alternatively, recent single neuron projectome data support our finding that hippocampal output neurons can be highly collateralized. Using sparse labeling and fMOST‐based whole‐brain reconstruction, Qiu et al. (2024) showed that individual hippocampal neurons send axon collaterals to multiple intra‐ and extra‐hippocampal targets. This agrees with our finding that AHN‐projecting ventral subicular neurons are not organized as a simple point‐to‐point pathway, but as a collateralized projection system. Thus, Qiu et al. (2024) provide independent single‐neuron‐level support for hippocampal collateralization, whereas our study defines the collateral architecture of a specific AHN‐projecting ventral subicular population.

Our findings indicate that ventral subicular‐projecting cells exert a wide‐ranging influence across brain areas, placing them in a unique position to shape complex, integrated responses. Specifically, as presently suggested, ventral subicular cells that project to the AH, driving defensive and anxiety reactions, may simultaneously impact a range of functions, including memory, motivation, arousal, circadian rhythms, neuroendocrine control, and the expression of other goal‐oriented behaviors. Further experimental evidence is certainly needed to prove this hypothesis. Finally, given the complex role of subicular cells in coding for spatial cues and for stressful and fearful events (Ciocchi et al. 2015; Forro et al. 2022; Jimenez et al. 2018; Lipski and Grace 2013; Malagon‐Vina et al. 2023; Poulter et al. 2021), it is critical to understand how this particular group of ventral subicular anterior hypothalamic‐projecting cells responds to actual and potential threatening stimuli, integrating their emotional valence and environmental location.

## Author Contributions

F.F.M., K.D., and N.S.C. conceived the project, designed the experiments, and wrote the manuscript. K.D. performed stereotaxic surgeries for viral tracer injections. F.F.M., K.D., and I.D.T. performed perfusion, tissue processing, and immunofluorescent procedures; F.F.M. prepared the figures; F.F.M. and N.S.C. performed the anatomical analysis; N.S.C. led funding acquisition and supervision.

## Funding

This work was supported by Fundação de Amparo à Pesquisa do Estado de São Paulo (2022/14359‐0, 2021/01642‐2, 2025/14464‐6, and 2024/12092‐1), Conselho Nacional de Desenvolvimento Científico e Tecnológico (303378/2024‐7 and 150992/2022‐0), Coordenação de Aperfeiçoamento de Pessoal de Nível Superior (88881.233817/2025‐01).

## Conflicts of Interest

The authors declare no conflicts of interest.

## Supporting information


**Figure S1:** Distribution of labeled cells in a Ai 6 female mouse after retrograde Cre insertion in the anterior hypothalamic region. (A) Schematic representation of the experimental design. (B) Injection site illustrating the AAVrg‐hSyn‐Cre‐WPRE‐hGH+ Fluoro Gold (FG) injection centered in the anterior hypothalamic nucleus (experiment FE#06). (C)–(F) Series of fluorescent microphotographs showing frontal sections arranged from rostral to caudal illustrating the distribution of ZsGreen1 fluorescence in the subicular fields following Cre‐mediated recombination. For abbreviation see list.

**Figure S2:** Experimental investigation of the projections from the SUB in female mice. (A) Schematic representation of the intersectional approach of experimental design. (B) Schematic representation showing the distribution and extent of the injection sites in female mice located in rostro‐caudal extent of the ventral SUB. Numbers on the top right of each section depict the approximate distance from the bregma. For abbreviations see list.

**Figure S3:** Projections from the rostral two‐thirds of the ventral SUB in female mice. (A)–(N) Fluorescent microphotographs showing a series of frontal sections arranged from rostral to caudal illustrating the distribution of mRuby anterogradely labeled fibers in experiment FE#6, for which the injection site is illustrated in Figures S2B and S3L. For abbreviations see list.

**Figure S4:** Projections from the caudal part of the ventral SUB in female mice (experiment FE#3). (A,B) Fluorescent microphotographs showing the extent of the injection site in the ventral SUB. (C) Injection of the AAVrg‐hSyn‐Cre‐WPRE‐hGH+ Fluoro Gold (FG) centered in the anterior hypothalamic nucleus. (D)–(H) Fluorescent microphotographs showing a series of frontal sections arranged from rostral to caudal illustrating the distribution of GFP anterograde labeling in the ventromedial hypothalamic nucleus (D), ventral premammillary nucleus (E), ventral tegmental area and compact part of substantia nigra (F), periaqueductal gray and pedunculopontine nucleus (G), and laterodorsal tegmental nucleus (H). For abbreviations see list.

**Figure S5:** Comparison between the projections from the rostral two‐thirds (experiment MAvr #24) and caudal third (experiment MAc#17) of the ventral SUB. (A) Fluorescent microphotograph of the injection site placed in the rostral two‐thirds of the ventral SUB (experiment MAvr #24). (B) Fluorescent microphotograph of the injection site placed in the caudal third of the ventral SUB (experiment MAc#17). (C)–(T) Paired comparisons between the projection fields from injections placed in the rostral two‐thirds (left column) and caudal third (right column) of the ventral SUB. Left column—Fluorescent microphotographs showing a series of frontal sections arranged from rostral to caudal illustrating the distribution of GFP anterogradely labeled fibers in experiment MAvr #24. Right column—Fluorescent microphotographs showing a series of frontal sections arranged from rostral to caudal illustrating the distribution of GFP anterogradely labeled fibers in experiment MAc#17. For abbreviations see list.

## Data Availability

The data that support the findings of this study are available on request from the corresponding author. The data are not publicly available due to privacy or ethical restrictions.
